# Glutamate transporter genes are associated with schizophrenia in South Indian population

**DOI:** 10.1186/1755-8166-7-S1-P42

**Published:** 2014-01-21

**Authors:** Ajay Jajodia, Harpreet Kaur, Ruchi Baghel, Gurpreet Kaur, Sanjeev Jain, Ritushree Kukreti

**Affiliations:** 1 CSIR-Institute of Genomics and Integrative Biology, Mall Road Delhi, India; 2 NIMHANS, Honsur Road, Bengaluru, India

## Background

Schizophrenia is a neurodevelopmental disorder and is manifested by disruption in cognitive ability along with positive and negative symptoms. Neurodevelopment abruptions involve pathologic processes caused due to genetic and environmental factors. Study aims to evaluate the association between the genetic polymorphisms of the neurodevelopmental gene and the risk of schizophrenia.

## Method

The study includes 482 schizophrenia cases and 401 age, sex matched controls. Genotyping was performed for single nucleotide polymorphisms (SNPs) of genes by primer extension reaction followed by the MALDI-TOF mass spectrometry (Sequenom^TM^). Polymorphisms involved in neurodevelopment processes like synaptic plasticity, synaptogenesis, signal transduction and activity of receptors/transporters. Genotypic tests were applied to examine the association of SNPs with disease. SNPs with p value < 0.1 were investigated by haplotypes analyses. Further, p values were adjusted with clinical variables using multivariate logistic regression. To evaluate interaction between the loci multifactor-dimensionality reduction (MDR) test was performed. Test for multiple corrections was applied using 10000 MaxT permutations.

## Results

Single locus association analysis showed association of rs2033267 (SLC1A3) OR=3.08, 95%-CI=1.98-4.77, rs10430590(PIP4K2A) OR= 1.71, 95%-CI=1.29-2.26. We identified a significant three marker haplotype of SLC1A3 significantly associated with disease with OR=3.81, 95%-CI=2.03-7.16. MDR analysis reveal interaction between rs2494750 and rs3803300 of v-AKT murine thymona viral oncogene homolog 1(AKT1) and rs16917204, rs56164415 of Brain Derived Neurotrophic Factor (BDNF) with cross validation 8/10 and Pvalue 0.0001,OR=13.07,95%-CI=8.97-19.03.

## Conclusions

We report a association of SLC1A3(5p13) with risk of schizophrenia development, which codes for glutamate transporter found in glial cells that functions to regulate neurotransmitter concentration at excitatory synapse. In addition interaction study revealed AKT1 and BDNF genes both having neuroprotective role.

